# The Prevalence of Adverse Childhood Experiences Among Children and Adolescents Who Display Harmful Sexual Behaviour: A Review of the Existing Research

**DOI:** 10.1007/s40653-022-00444-7

**Published:** 2022-07-29

**Authors:** Dulcie Faure-Walker, Nigel Hunt

**Affiliations:** 1 National Clinical Assessment and Treatment Service (NCATS), The National Society for the Prevention of Cruelty to Children (NSPCC), London, UK; 2 Faculty of Medicine & Health Sciences, University of Nottingham, Nottingham, UK

**Keywords:** Children, Adolescents, Adverse childhood experiences, Sexual behavior, Sexual abuse, Physical abuse, Emotional abuse, Neglect, Domestic violence, Trauma

## Abstract

There is no systematic review focusing on the prevalence of adverse childhood experiences (ACEs) among children and adolescents who display harmful sexual behaviour (HSB). This study addresses this gap to further our understanding of the aetiology of HSB among children and adolescents. The full text of 87 articles was retrieved and assessed for eligibility, following which 10 articles were deemed relevant for inclusion in the review. These 10 studies were then subjected to quality assessment, data extraction and synthesis. The present review included only studies that used data pertaining to both males and females, and some studies provided a comparison between males and females. There were higher rates of sexual victimisation among females who display HSB. Additionally, child sexual abuse perpetrated by female caregivers is likely to be higher than most of the existing body of research suggests. The findings of the present review corroborate many of the hypotheses discussed in the introduction, emphasising that children and adolescents who display HSB are more likely to have come from backgrounds of trauma, signaling the importance of multi-agency responses, early intervention and the importance of protective factors.

## Introduction

### Background

Research into the perpetration of sexual harm has shifted from an adult focus to include adolescents and children (Hawkes, 2011), with harmful sexual behaviours (HSBs) employed as a term to refer to this behaviour among those aged under eighteen. Children and adolescents who display such behaviours are more likely to have experienced adverse childhood experiences (ACEs) in some capacity, including physical, emotional or sexual abuse, neglect, family break-down/bereavement, witnessing and/or experiencing domestic violence, and parental substance abuse (Hackett, 2014; Hawkes, 2009; Rich, 2011). Although this research has led to an improvement in the recognition of, and practice in response to, this population, there is still much to be learnt about the aetiology and manifestation of HSB. The present study aims to address this gap in the literature to further our understanding of the trajectory of HSB among children and adolescents.

### Defining Adverse Childhood Experiences

Adverse childhood experiences (ACEs) are defined as stressful experiences that require significant psychological, social and neurodevelopmental adaptation by the developing child (Felitti et al. 1998; Lacey & Minnis, 2019; McLaughlin, 2016). When early experiences incur serious physical and/or psychological harm and stress, an individual can develop dysfunctional or problematic views including beliefs about themselves, others and the world around them (Streeck-Fischer & van der Kolk, 2000).

Felitti and colleagues’ (1998) study, from which the ACE questionnaire was formed, proposed 10 categories of childhood adverse experiences. The categories include emotional, physical and sexual abuse, neglect, family separation, breakdown or bereavement, witnessing domestic violence, parental substance misuse, poor parental mental health, family history of offending, and other negative childhood experiences. These categories are helpful in conceptualising the broad range of traumatic early experiences and have been utilised in studies seeking to explore possible consequences of adversity in childhood (Flaherty et al., 2013; Hillis et al., 2004; Levenson, Willis & Prescott, 2016). More recently there has been a focus on how to prevent adverse experiences in childhood as studies have evidenced how they can lead to long-term psychological and physical health impairments (Hughes et al., 2017), and have been associated with trajectories of offending (Fox et al., 2015). The findings of the current study will be summarised in the context of these 10 categories.

### Characteristics of Young People with HSB

It is widely accepted that the healthy sexual development of children and adolescents who display HSB has been compromised in some way (Salter et al., 2003). Almond, Canter & Salfati (2006) distinguish three categories of children and adolescents who sexually harm: ‘abused’, ‘delinquent’ or ‘impaired’. ‘Abused’ can relate to abuse by family members, friends or others. ‘Delinquency’ involves variables associated with antisocial behaviour or criminality, such as property offences, bullying others and substance abuse. ‘Impaired’ incorporates variables that potentially impair a young person’s capability to develop a healthy sexual understanding, including educational difficulties, special educational needs and social isolation. More recently, studies have focused largely on the vulnerability of most children and adolescents who display HSB, with a focus on prevention and education as opposed to stigmatisation and criminalising. Approximately 30–50% of HSB involves other young people as perpetrators (Campbell, Booth, Hackett & Sutton, 2018). In their review of the literature on children with HSB, Malvaso, Proeve, Delfabbro & Cale (2020) found evidence that disrupted patterns of attachment, particularly anxious attachment, played a role in the manifestation of HSB; with particular consideration of the internalisation of trauma from early years and how this should be a focus of interventions.

### Purpose of the Current Review

The purpose of this study is to address the existing gap in research through systematically reviewing the research on the prevalence of ACEs among children and adolescents who display HSB, including a focus on sex differences. There is no recent review on the subject, so it will provide a comprehensive and critical analysis of the current knowledge on this topic and will enable recommendations to be made for future research and practice.

## Method

### Search Strategy

Scopus, ScienceDirect, Web of Science, PubMed, Social care online and ASSIA were systematically searched in December 2020. The following terms were used to search for relevant literature: (harmful sexual* OR inappropriate sexual* OR abusive and sexual*) AND (behaviour* OR behavior*) AND (child OR infant OR adolescent) AND (life change events OR emotional abuse OR child abuse OR physical abuse OR sexual abuse OR psychosocial stress OR childhood adversity OR child maltreatment OR neglect OR parental substance misuse OR parental substance abuse OR family in prison* OR bereavement OR parental mental illness OR family breakdown).

The citation, author, title, year and abstract of each study was assessed to determine if the subject is relevant. If deemed relevant the full paper was obtained. The full papers were then read in line with the inclusion/exclusion criteria outlined in Table 1 to determine which papers should be included.

**Table 1:** Inclusion and exclusion criteria

	Inclusion		Exclusion	
Population	Children and adolescents (< 18 years) who have been exposed to any category of ACEs Studies focusing on males *and* females		Post-adolescent ACE exposure Adult men and women (=/> 18 years) Studies focusing solely on males *or* females	
Exposure	Any exposure to ACE/s, based on the categories of the Adverse Childhood Experiences Questionnaire, which are: 1). Emotional abuse 2). Physical abuse 3). Sexual abuse 4). Emotional neglect 5). Physical neglect 6). Family separation/breakdown/bereavement 7). Domestic violence 8). Substance misuse/abuse 9). Poor parental mental health 10). Family member in prison		No exposure to ACE/s No HSB	
Comparator Outcomes	No comparator or other types of adverse experience Sufficient evidence of harmful sexual behaviour from self-disclosure, parents/guardians, police, health care professionals or other professionals involved in the young person’s care		N/A Post-adolescent HSB.	
Context	Community, secure setting, residential, young offenders’ institution, hospital wards English language articles		Non-English articles	
Study design	Cohort, case control and cross sectional studies		Reviews, opinion papers	

### Search Results

**Fig. 1:**
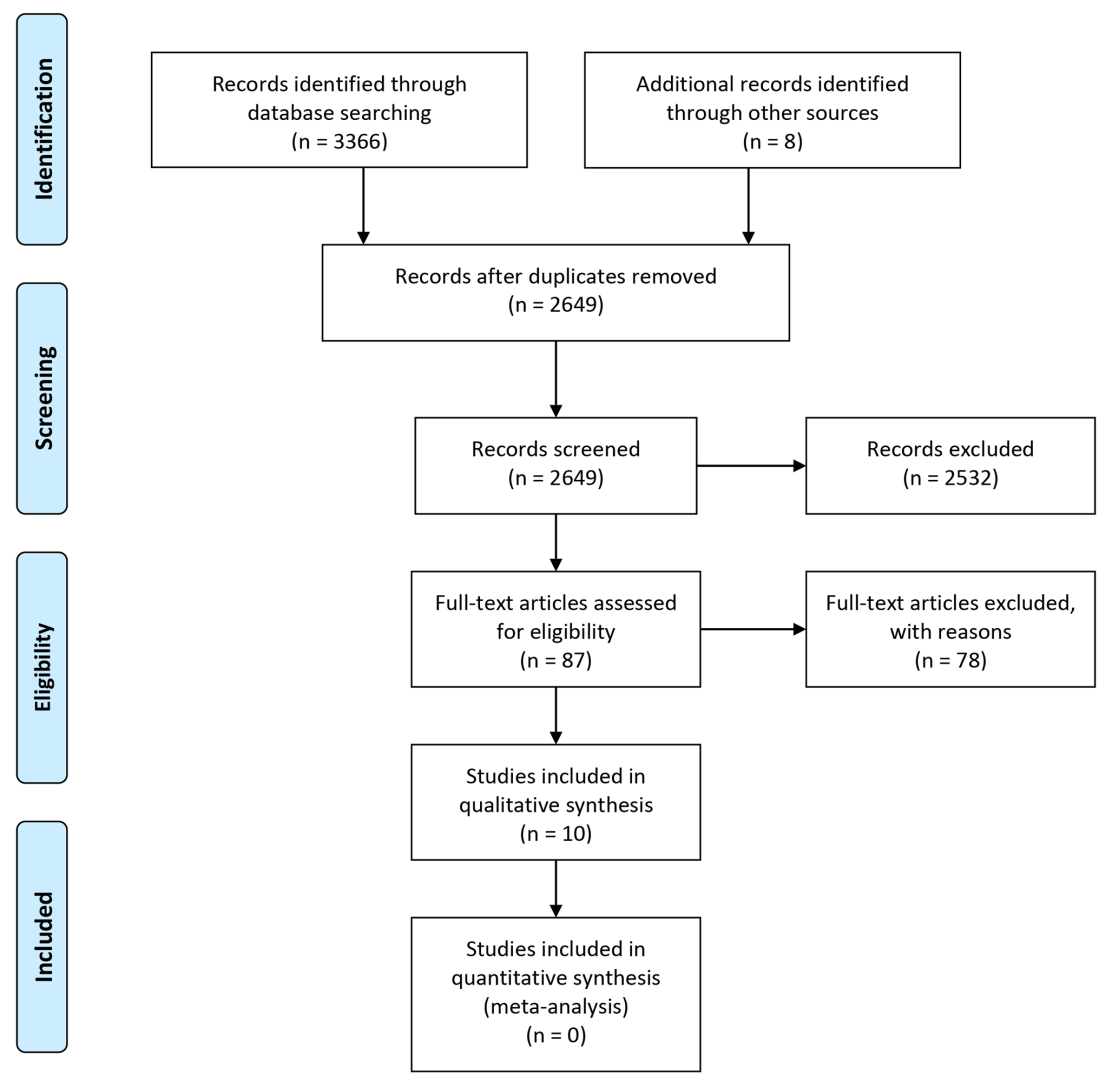
PRISMA flow template: selection process

The searches produced 3374 hits in total. This was reduced to 10 that were subjected to quality assessment, data extraction and synthesis (see Fig. 1 for details).

### Data Extraction

Using a pro-forma, the following data were extracted by the author: objectives, sample size and demographics, sampling method, country of publication, category of ACE/s, incidence of sexualised behaviour and findings.

## Results

See Table 2 for characteristics of the included studies. It is not possible to determine how many participants were children, adolescents or young adults as on most occasions the authors did not say. All the studies were published in English. Seven studies had participants from the UK, and three from the USA. The headings that follow use the 10 categories of ACE described in the Introduction. They are in no particular order as it is not possible to determine prevalence or chronology.

**Table 2:** Characteristics of included studies

Study reference, location of study and design	Setting/context	Sample size (*n*)	Gender	Age range	Objective	Findings
Balfe, Hackett, Masson & Phillips, 2019 England and Wales Retrospective cohort Bladon, Vizard, French & Tranah, 2005 England Cross-sectional	9 ‘child welfare’ HSB services between 1992–2000 Community forensic treatment service	117 141	Male and Female* Male (*n* = 130, 92.2%) Female (*n* = 11, 8%)	10–17 5–21	To provide insight into the family and social contexts of young people with HSB To investigate the nature and prevalence of psychopathology in a cohort of children and adolescents displaying HSB	Erratic living situations, poor family relationships, unstable parental backgrounds, social and educational difficulties. Over half had experienced a form of abuse other than sexual abuse, and just over half had been sexually abused or were suspected of being sexually abused. Significant psychosocial and psychiatric vulnerabilities including high prevalence of sexual and physical abuse and frequent diagnoses of posttraumatic stress disorder and conduct disorder.
Hackett, Masson, Balfe & Phillips, 2013 England Cross-sectional	Community treatment service	700	Male (*n* = 676, 97%) Female (*n* = 24, 3%)	5–28	To present the individual, family and abuse characteristics of a cohort of children and adolescents displaying HSB	High rates of sexual and non-sexual victimisation. The most common age at referral was 15, though a third of all referrals were for children aged 13 or under. Over a third of the sample were learning disabled. Victims were usually known to the perpetrator but in 75% of cases were not related. Just over half of the sample abused females only, but 49% had at least one male victim.
Hutton & Whyte, 2006 Scotland Cross-sectional	Community treatment services (national)	189	Male (*n* = 178, 94%) Female (*n* = 11, 6%)	5–20	To report characteristics pertaining to Scottish children and adolescents who display HSB	Disrupted childhoods of sample, including witnessing violence within the family and physical or sexual assault and disengaged from education. Indications that HSB manifests differently between males and females.
James & Neil, 1995 England Retrospective cohort	Community (postal survey)	34	Male (*n* = 31, 91%) Females (*n* = 3, 9%)	12–17	To estimate a 1 year prevalence of HSB among children and adolescents and present the sociodemographic and victimisation histories	Backgrounds of neglect, physical, and/or sexual abuse. Behavioural and psychological problems were common.
Manocha & Mezey, 1998 England Cross-sectional	Assessment and treatment centre	51	Male (*n* = 49, 96.1%) Female (*n* = 2, 3.9%)	13–17	To describe the characteristics of a cohort of sexually abusive youth	Prior histories of abuse and victimisation and lack of protective parenting among sample. Authors suggest young people with harmful sexual behaviour may have experienced environmental, familial, interpersonal and developmental difficulties.
McClellan, McCurry, Ronnei, Adams, Storck, Eisner & Smith, 1997 America Retrospective cohort	Tertiary psychiatric hospital	499	Males (*n* = 314, 63%) Females (*n* = 185, 37%)	5–18	To examine the differences in abuse histories and the development of HSB in a sample of children and adolescents with mental health difficulties	Females in sample were more likely to have been sexually abused, and their abuse histories were more severe. Males had a lower threshold of abuse exposure required to develop sexually inappropriate behaviours and were more likely to display victimising behaviours.
Montgomery-Devlin, 2004 Northern Ireland Cross-sectional	Community based treatment project	71	Male and female*	12–18	To examine the characteristics of children and adolescents referred to the project and behaviour that led to referral	Sample experienced considerable disruption in their lives, including domestic violence and physical or sexual abuse.
Ryan, et al, 1996 United States of America Cross-sectional	The National Adolescent Perpetrator Network (NAPN)	1616	Males (*n* = 1574, 97.4%) Females (*n* = 42, 2.6%)	5–21	To describe the sociodemographic factors of young people with HSB	Physical and sexual abuse, neglect, and loss of a parental figure were common among the sample’s histories. Approximately a quarter of the sample who had been victims of sexual abuse reported that the perpetrator was female, and the vast majority of offences perpetrated by the sample involved female victims.
Vizard, Hickey, French & McCrory, 2007 England Cross-sectional	Community NHS specialist service	280	Males (*n* = 256, 91.5%) Females (*n* = 24, 8.5%)	5–21	To describe the psychosocial and behavioural characteristics of young people with HSB	Prevalence of developmental risk factors among sample, including extremely emotionally neglectful and abusive backgrounds, family instability and dysfunction, neuropsychological deficits and mental health problems.

* Approximately 95% of the 117 cases were male and ‘white’, but the authors do not specify other categories.

**The author does not specify the number of males and females in the sample, but states that the majority of were male.

### Emotional Abuse

Five studies discuss emotional abuse. Varying amounts of emotional abuse were found. The figures varied from 74% (Vizard et al., 2007), 68.1% (Bladon et al., 2005), 61.2% (James & Neil, 1995) and 50% (Hutton & Whyte, 2006). The lowest figure was 13.7% (Manocha & Mezey, 1998), though they also noted that 29.4% described their caregivers as ‘rejecting’, ‘uncaring’, ‘unloving’ or ‘disinterested’. It is not clear whether such perceptions are indicative of emotional abuse or neglect.

### Physical Abuse

Nine studies discuss physical abuse. Varying amounts of physical abuse were found. These varied from 66% (Vizard et al., 2007), 51.8% (Bladon et al., 2005), 41.9% (James & Neil, 1995), 37% (Hutton & Whyte, 2006), to 31% (Montgomery-Devlin, 2004). McClellan et al. (1997) compared males and females, with 64% of males being physically abused, and 67% of females. Ryan et al. (1996) note that at the point of referral, and prior to potential further disclosures made by patients during the treatment process, it was known that 41.8% (*n* = 675) had been victims of physical abuse. This indicates a possible difference in reporting among studies.

Manocha and Mezey (1998) found regular parental violence towards the child or adolescent to have been present in 23.5% (n = 12), but this is not termed as physical abuse per se by the authors. They also note that sibling violence was reported in 9.8% (n = 5) of cases. Balfe et al. (2019) do not specifically measure physical abuse, however the authors note how physically abusive behaviour was displayed towards the sample under the guise of chastisement. They associated this with caregivers unable to control their children and appropriately discipline and set boundaries for children within their care.

### Sexual Abuse

All ten studies discuss sexual abuse. Varying amounts of sexual abuse were found. These ranged from 71.6% (Bladon et al., 2005) and 71% (Vizard, 2007), to 50% (Hackett et al., 2013), 35.4% (James & Neil, 1995) and 29.4% (Manocha & Mezey, 1998). Ryan et al. (1996) note that at the point of referral, prior to potential further disclosures made by patients during the treatment process, 39.1% had been victims of sexual abuse.

Hutton & Whyte (2006) found the prevalence of known sexual abuse among their sample to be 19%, but they also measure suspected sexual abuse among a further 31%. Balfe et al. (2019) distinguish between intra and extrafamilial sexual abuse. 14% had disclosed being sexually assaulted by a father/stepfather, 8% by an uncle or grandfather and 4% by older male relations such as cousins. Some children were abused by more than one family member. 9% of the children reported being sexually assaulted by female relations, including their mothers. 13% had been sexually abused by extrafamilial young people, mainly by older boys.

Comparing males and females, McClellan et al. (1997) found a Fig. 80% among the females in their sample, but only 40% among males. Additionally, the authors found that females had more severe sexual abuse histories than males, with higher rates of abuse by intercourse and multiple perpetrators.

As well as sexual abuse, Manocha and Mezey (1998) also describe a ‘lack of sexual boundaries’, with 15.7% of children and adolescents in their study disclosing that they had frequently witnessed sexual acts between their parents of caregivers. 33% of their sample had regular access to sexually explicit material within their family home, although the nature (e.g. legality) of this material is not recorded.

### Neglect

Eight studies discuss neglect. Unmet physical and psychological needs were a prevalent concern across the included studies. The highest prevalence (61.2%) of neglect was found by James and Neil (1995), followed by 59% for Vizard et al. (2007), 58.9% (Bladon et al., 2005), and 45% (Hutton & Whyte, 2006) who also measured parental rejection, which was experienced by 43%. Ryan et al. (1996) reported that at the point of referral 25.9% of the sample were subject to neglect. The prevalence of neglect to be slightly higher among males (31%) than females (26%) (McClellan et al., 1997).

Balfe et al. (2019) describe “profound” levels of emotional abuse (by parents, relatives, stepparents or carers) among 16% of their sample. Some children were subject to child protection concerns or plans, with 25% described as suffering from general neglect or poor levels of care. Unmet physical and psychological needs were a prevalent concern among the young people.

The lowest prevalence was found in Manocha & Mezey (1998), where neglect was found to have been experienced by 11.8% (*n* = 6) of their sample. As previously reported, the authors also note that a third of the children and adolescents in their sample described their caregivers as ‘rejecting’, ‘uncaring’, ‘unloving’ and ‘disinterested’.

### Family Separation, Breakdown or Bereavement

Eight studies discuss this topic. The breakdown of families and high levels of social care involvement were recurrent themes in the included studies. Bladon et al., (2005) found that 39% of their sample were on full care orders (with the local authority holding most of the responsibility over the child/adolescent). 34% of the sample had experienced multiple (at least three) placements. Hackett et al. (2013) found that 14% of their sample were on full care orders, with 6% in secure accommodation as a result of their HSB. James & Neil (1995) found that 42% of their sample were not in the care of their parents (16.1% adopted, 3.2% fostered, 9.7% accommodated by social services, 12.9% in the care of social services).

Hutton & Whyte (2006) found that 54% of their sample’s parents had separated. They also measured the experience of bereavement in childhood (although not necessarily of a parent), which was experienced by 15% of their sample. Ryan et al. (1996) found that 13.6% had suffered a bereavement, and 57% had experienced the loss of a parental figure, of which 12% resulted from the death of one or both parents, and 34.2% from the child or adolescent being removed from the care of their parents. Vizard et al., (2007) found that 73%of their sample had experienced loss of their parents, either through parental separation or bereavement. This was the case with 41% of Balfe et al.’s (2019) study, with parental separation among 31% of young people, and parental death among 10%. Balfe et al. (2019) note that the information they could access in their file review was limited, but 17% of young people were “living in care” at the time of referral. There were no further details about this. A further 15% lived in children’s homes, 13% in foster care, and 8% in secure units. Most of the young people in their sample had unstable and unpredictable living situations.

At the time of referral, Manocha & Mezey (1998) established that 31.4% of their sample lived at home with both biological parents, 21.6% were living with one biological parent and one stepparent, 13.7% came from single-parent households, which was higher than the national average (Rabindrakumar, 2013). Out of the 23.6% who were not in the care of their biological parents, 9.8% lived with foster parents, 5.9% with grandparents or other relatives, 5.9% with multiple caregivers, 2% with no parental figure and 13.7% had lost one or more biological parents through death. Montgomery-Devlin (2004) reports similar figures, with 33% in Belfast and 16% in Derry not living with their immediate family at the point of referral.

### Witnessing Domestic Violence

Six studies discussed domestic violence. Those that did include the variable of violence within the family home concluded that it was common. The highest prevalence was found in Ryan et al.’s (1996) study, where 63.4% of their sample had witnessed violence within their family home. This was followed by 49% (Vizard et al., 2007), 40% (Hutton & Whyte, 2006), 33% (Montgomery-Devlin, 2004), and 26% (Balfe et al., 2019), though the last described the violence as ‘extreme’ in some cases. Manocha & Mezey (1998) recorded marital (as opposed to domestic) violence, reporting a figure of 373%.

### Parental Substance Misuse

Four studies discussed parental substance misuse. The highest reported figures were 57% of males and 69% of females (McClellan et al., 1997) came from families where there was a history of substance abuse among family members. Lower figures of 33% (Hutton & Whyte, 2006) 27.9% (Ryan et al., 1996) and 22% (Balfe et al., 2019) were also reported.

### Poor Parental Mental Health

The only study to include parental mental health as a variable was McClellan et al. (1997). They differentiate between ‘mood disorders’ and ‘psychotic disorders.’ Mood disorders were prevalent among 30% of males and 45% of females. For psychotic disorders, the prevalence was 12% among males and 6% among females.

### Family History of Offending

Three studies included information about their sample’s family history of offending. Vizard et al., (2007) found that 28% had a convicted ‘Schedule One’ offender (convicted of an offence against a child) within their family. McClellan et al. (1997) and Manocha & Mezey (1998) did not record such specificities. The former study uses the variable of ‘antisocial histories’, as opposed to family criminality. This does not necessarily mean the child or adolescent’s caregiver was involved with the criminal justice system. They found antisocial histories prevalent in 46% of males and 51% of females in their sample. Manocha & Mezey (1998) found that parental criminality existed in 27.5% of their cases, including three instances where the child or adolescent had a father or stepfather in prison at the time of data collection.

### Other Negative Childhood Experiences

Vizard et al., (2007) recorded that 44% of the children and adolescents in their study experienced what they deemed to be ’inappropriate sexual boundaries’ within their family home. As their study was based on case file reviews rather than on their definitions, such terminology is ambiguous and potentially subjective.

Other studies note complex histories of social care involvement. Montgomery-Devlin (2004) found that 38% of her sample were subject to child protection planning at the time of referral to their project. Manocha & Mezey (1998) established that 27.5% of their sample been placed on the child protection register at some point in their lives, including 7.8% who were on the register at the point of referral. There were 21.6% who had experienced numerous care placements. Child protection investigations or criminal proceedings occurred in 84.2% of cases at the time of the study. Vizard et al. (2007) reported that 64% of children and adolescents were subject to child protection planning. In Balfe et al.’s (2019) study, there had been previous concerns raised in 91% of cases, with only 9% described as supportive and stable environments. Ryan et al. (1996) note that only a minority of the disclosures made by children and adolescents resulted in prosecutions (16.9% of physical abuse and 37% of sexual abuse disclosures).

## Discussion

### Findings of the Review

This is the first review where the research on the prevalence of different types of ACEs among children and adolescents with HSB has been systematically searched and appraised. The review demonstrates that children and adolescents who display HSB are likely to have experienced multiple types of adversity. In each included study, most of the children and adolescents with HSB had come from backgrounds where their healthy sexual development had been compromised in some way. While not all studies discussed each form of ACE, some ACEs were discussed by the majority. These included physical abuse, sexual abuse, neglect, family separation, breakdown or bereavement, and witnessing domestic violence.

Gender differences was a recurrent theme. The present review included studies that used data pertaining to both males and females combined, although some studies provided a comparison between them. Generalisations should be made with caution, as the female samples were typically much smaller than the male counterparts, however the present review found that more complex victimisation histories could be found among females. For example, Hackett et al. (2013) found significantly higher rates of sexual victimisation among the females in their sample than the males, corroborating McClellan et al. (1997) who found that not only were females with HSB much more likely to have been sexually abused but tended to have more severe sexual abuse histories than males, with higher rates of abuse by intercourse and multiple victimisers. Such findings indicate that it would be helpful for comparative studies in order to elucidate possible gender differences in both the prevalence of adverse experiences and patterns of sexual harm, echoing previous researchers including Hallett, Deerfield and Hudson (2019), who call for exploration of the role of gender, gendered understandings and gendered trajectories of HSB. The literature on females who sexually harm is sparse in comparison to males, and in order for assessment and intervention to be effective, research is needed to elucidate the needs of this population.

### Theoretical and Methodological Issues

The review raised several theoretical and methodological issues. The key theoretical issue is the difficulty defining the constructs used, which raises issues regarding whether the ten categories are appropriate. There are several examples of ambiguity. For instance, what should we mean by neglect? There are several categories which may be considered neglect. Parental substance abuse is one possibility, as is a family history of offending, poor parental mental health, or family separation, breakdown or bereavement. It is not necessarily that these lead to neglect, but it is probable that at least on some occasions the consequences of these will meet the threshold for neglect.

In the United Kingdom, neglect tends to be the most commonly cited concern in child protection (Radford et al., 2011). One potential reason for this is because it signifies a persistent failure to meet a child’s basic physical and psychological needs (Department for Education, 2018), a broad definition. At the same time, however, neglect may be experienced alongside these other harms that make it difficult to identify as well as prove legally (Brandon, Bailey, Belderson & Larsson, 2014; Glaser, 2002). We must therefore bear in mind the likely gap between the level of neglect occurring and that that comes to the attention of services.

Another problem is that of defining abuse. In the present study we have separated emotional, physical and sexual abuse, but these often overlap. ACEs are interconnected rather than occurring independently of each other (Dong et al., 2004). It is not always easy to clearly distinguish emotional, physical and sexual abuse from each other; sexual abuse is often emotional abuse, as is physical abuse. Each is unlikely to occur in isolation. Rees (2010) comments that emotional abuse is often considered to lack the gravitas when compared to physical and sexual abuse, compounded by difficulties in its recognition, definition and proving its occurrence, however it is at the centre of these experiences and may be its most damaging element. The difficulties in defining abuse can be demonstrated in sibling sexual abuse cases, which have been historically unrepresented in the literature due to the misconceived idea that is a form of sexual exploration and less harmful (Yates, 2017), however often have added layers of potential harm such as secrecy and barriers to disclosure and help.

Instead of trying to define ten separate categories which clearly overlap in many ways it might be more productive to develop a theoretical framework that has a key focus on two aspects: abuse and neglect. Abuse includes emotional, physical and sexual abuse, while neglect focuses on the other aspects, including family separation, breakdown and bereavement, witnessing domestic violence, parental substance misuse, poor parental mental health, and a family history of offending. This approach would enable a focus on the key differences between actual abuse and neglect, and the differential impact these may have on development and later offending behaviour.

If the categories are difficult to define theoretically then it is not going to be possible to assess them methodologically. Many of the studies failed to provide any clear definitions of the concepts they were measuring. The categories cannot be measured without a clear definition. Future research should aim to focus on a clearer theoretical typology of ACEs. It is not just about a reduction to two fundamental categories of abuse and neglect, but a clearer definition of the subcategories of ACEs. For instance, Finkelhor (2018) postulates that the current ten item typology requires the inclusion of a more comprehensive range of adversities. Skinner et al. (2021) also raise points pertaining to the importance of considering contextual factors, such as socioeconomic circumstances.

To focus on a more specific category, which also raises a difficulty for the search process, it is a challenge to find a global consensus for the terminology referring to sexual abuse carried out by children and adolescents. Preliminary searches of the ‘grey literature’ produced by the charity sector that work with child and adolescent perpetrators of sexual abuse and produce briefings and publications on this area (inter alia NSPCC, Barnados, Stop It Now) used the term ‘harmful sexual behaviour’, defined initially by Hackett (2014). This is not the same phrase used in other countries, a difficulty also noted by Smith et al. (2014). Although the searches included syntax variations, there is the possibility that studies from other countries were not found as they used different terminology to refer to sexual abuse carried out by children and adolescents.

Additionally, only English language studies were included in the review due to time and resource constraints. Including non-English studies would provide a much more robust picture of the global prevalence of ACEs among children and adolescents who sexually abuse. Despite an exhaustive search, most of the included studies were from England (6 out of 9). This may be attributed to the defining terms of ‘adverse childhood experiences’ and ‘harmful sexual behaviour’, which may differ from the terms used in different countries. Thus, studies using different terminology may not have been located during the search process.

## Conclusions and Recommendations

The findings of the present review emphasise that children and adolescents who display HSB are more likely to have come from backgrounds of trauma, both abusive and neglectful, though there is a distinction between correlation and causation. As Masson, Hackett, Phillips and Balfe (2015) note, it is more helpful and hopeful to consider ACEs as markers of vulnerability as opposed to risk factors. It is much more helpful and hopeful to understand this population as requiring protection, treatment and education rather than criminalisation. There are many intervening events and variables that mediate childhood exposure and later problems. It appears that some children are provided with the support or are equipped with the resilience which serves to mitigate the harm caused by ACEs. Additional research on children who experience such adverse early experiences but do not go on to abuse others may be helpful in helping policy makers and practitioners improve outcomes for children and adolescents with these trauma histories.

Interventions for young people with HSB have largely evolved from those developed for use with adult perpetrators. In response to the limited evidence base for the appropriateness of this, Campbell et al.’s (2018) review found that promoting the role of parents or carers, considering the environmental context of the young person, and equipping young people with interpersonal skills as well as knowledge were critical components of successful interventions for young people with HSB. This must be underpinned by a therapeutic relationship built on trust, where a young person feels safe. Considering the prevalence of ACEs the present study revealed, ensuring feelings of safety for a young person during intervention is imperative. This refers to psychological and physical safety, and consideration of a young person’s wider context (including their housing situation). The sequencing of interventions in important; intervention while a young person’s basic needs are not being met is unlikely to be effective. Their capacity to engage will be hindered if they are consumed by trying to manage danger to survive day to day.

An interesting area for future research is how specific adverse experiences are associated with different types of HSB. McClellan et al. (1997) found that a history of neglect was associated with what they term as sexually reactive behaviours (responses which indicate maladaptive coping mechanisms or developmentally inappropriate behaviours) and victimising behaviours (which are more controlling, coercive and threatening). They also found that among the males in their study, HSB was associated with higher rates of sexual abuse perpetrated by mothers or stepmothers. This finding is important to consider alongside the suggestions that child sexual abuse perpetrated by female caregivers is likely to be higher than the majority of the existing body of research suggests (Vandiver & Kercher, 2004; Wijkman, Bijleveld & Hendriks 2010), overlooked by many as it violates the societal expectations of women as caregivers and nurturers.

Importantly, as Browne & Hollin (1996) emphasize, while a cycle of abuse, referring to the transmission of abuse through generations in a likewise manner, may exist, this chain of events is not an inevitable one, signaling the importance of multi-agency responses, early intervention and the importance of protective factors. Those who display HSB are a heterogeneous population and the crossover of ‘victim’ to ‘perpetrator’ may not always be clear. Viewing HSB through the lens of trauma is important to identify vulnerabilities and areas of unmet need, with careful consideration of language due to the stigma and potentially lifelong harmful effects that these aforementioned labels carry.
